# Microrheological Characterization of Collagen Systems: From Molecular Solutions to Fibrillar Gels

**DOI:** 10.1371/journal.pone.0070590

**Published:** 2013-08-02

**Authors:** Marjan Shayegan, Nancy R. Forde

**Affiliations:** 1 Department of Chemistry, Simon Fraser University, Burnaby, British Columbia, Canada; 2 Department of Physics, Simon Fraser University, Burnaby, British Columbia, Canada; Massachusetts Institute of Technology, United States of America

## Abstract

Collagen is the most abundant protein in the extracellular matrix (ECM), where its structural organization conveys mechanical information to cells. Using optical-tweezers-based microrheology, we investigated mechanical properties both of collagen molecules at a range of concentrations in acidic solution where fibrils cannot form and of gels of collagen fibrils formed at neutral pH, as well as the development of microscale mechanical heterogeneity during the self-assembly process. The frequency scaling of the complex shear modulus even at frequencies of ∼10 kHz was not able to resolve the flexibility of collagen molecules in acidic solution. In these solutions, molecular interactions cause significant transient elasticity, as we observed for 5 mg/ml solutions at frequencies above ∼200 Hz. We found the viscoelasticity of solutions of collagen molecules to be spatially homogeneous, in sharp contrast to the heterogeneity of self-assembled fibrillar collagen systems, whose elasticity varied by more than an order of magnitude and in power-law behavior at different locations within the sample. By probing changes in the complex shear modulus over 100-minute timescales as collagen self-assembled into fibrils, we conclude that microscale heterogeneity appears during early phases of fibrillar growth and continues to develop further during this growth phase. Experiments in which growing fibrils dislodge microspheres from an optical trap suggest that fibril growth is a force-generating process. These data contribute to understanding how heterogeneities develop during self-assembly, which in turn can help synthesis of new materials for cellular engineering.

## Introduction

Collagen is the predominant structural protein in vertebrates, where it is responsible for tensile strength of connective tissues such as tendon, bone, skin and cartilage, and where it forms the basis for the extracellular matrix surrounding our cells. Collagen is also widely used as a biomaterial, where its uses span its incorporation into replacement tissues, products in the personal care industry, and substrates for cellular growth, differentiation and engineering. In all of these examples, collagen’s mechanical properties are of substantial importance: its elasticity and stiffness on the macroscale are important for materials performance, while on the microscale we are increasingly learning about the importance of mechanics in dictating cellular fate [1]–[7].

At the molecular level, collagen is a triple helix of polypeptide chains. Extracellularly, individual collagen proteins assemble hierarchically into fibrils, highly ordered structures of ∼100 nm in diameter and tens of micrometers in length [8]. Subsequent assembly of these fibrils into networks or fibres can follow, which, depending on tissue type, may also include organization of minerals or other protein components [9]. Collagen mechanics are coupled throughout its assembly hierarchy, implying that changes in molecular-level interactions can impact higher-level mechanical response [10]. The first stage of assembly, that of going from isolated triple helical proteins to fibrils, can be replicated *in vitro* through appropriate changes in solution chemistry. The resulting fibrils possess similar structural ordering as those found *in vivo*, suggesting that collagen’s specificity of self-association is encoded in its protein sequence, and that *in vitro* experiments can provide relevant insight into the process by which collagen self-associates *in vivo* [11].

Understanding the mechanism by which interactions between individual collagen molecules drive collagen assembly into fibrils is of key importance for targeting of physiological processes such as extracellular remodelling during development and cancer metastasis, keloid scar formation during wound healing and repair and regeneration of injured collagenous tissues such as tendon and cartilage. Additionally, rational design of the mechanics of collagen fibrils and networks *in vitro* will enable better design of matrices for cellular engineering.

A substantial body of work has been devoted to characterizing the kinetics of fibril formation and understanding the chemical basis for self-assembly [12]–[16]. More recently, the mechanics of collagen have been quantitatively studied at the molecular and fibrillar levels [10], [17]–[22], as have the mechanical properties of collagenous networks and their impact on cellular fate [1], [23]–[28]. Nonetheless, there remain to be answered substantial biophysical questions about the timescales, strengths and types of interactions between collagen molecules in solution, and the mechanism by which collagen molecules undergo ordered assembly into fibrils. Furthermore, whether microscale heterogeneity of mechanics observed in fibrillar networks [23], [26], [29] can be controlled and the importance of the range of mechanical environments sensed about a cell on timescales relevant to regulatory mechanisms and motility remain open questions.

In this work, we demonstrate the utility of optical-tweezers-based microrheology to probing the microscale viscoelastic response of collagen both in solutions of triple-helical collagen molecules and in gels of self-assembled collagen fibrils. By determining the local complex shear modulus, we find that solutions of collagen molecules exhibit homogeneous response, while mechanical heterogeneity at the microscale appears during early phases of fibrillar growth, developing further during this growth phase. Our experiments measure changes in frequency-dependent viscoelasticity over many orders of magnitude of frequency (timescales of interactions) while fibrils assemble. Previous dynamical measurements of rheological changes occurring during protein or peptide self-assembly have been limited to frequencies <100 Hz [24], [27], [30]–[33]; our optical-tweezers-based probe system extends the range of frequencies spanning from 1 Hz to >1 kHz, thus enabling study of interactions in more dilute systems and for more transient interactions. This dynamic range encapsulates timescales that are relevant for protein-protein interactions, molecular motor activity, protein-cell adhesion and cytoskeletal remodelling, which allow cells to sense, respond to and move through their external environments [5], [34], [35]. We demonstrate that the ability to probe over this broad range of timescales provides information about the time and concentration dependence of intermolecular collagen interactions. Our results correlate changes in viscoelastic properties with the kinetics of fibril formation in solution, suggesting that it is during the growth phase of fibril formation that mechanical heterogeneity develops on the microscale.

## Materials and Methods

### Materials Preparation and Characterization

Collagen type I (rat tail tendon) was purchased as a stock solution with a concentration of 5 mg/ml in 20 mM acetic acid (Cultrex Invitrogen), acidic conditions (pH = 3.3) in which collagen does not form fibrils. Concentrations below 5 mg/ml were obtained through dilutions in 20 mM acetic acid. Concentrations of collagen samples (stock and dilutions) were verified by ELISA [36]. Sample purity was verified by SDS-PAGE electrophoresis using both Coomassie staining and fluorescence labeling for visualizing collagen [37]. Sample monodispersity was probed using dynamic light scattering. These measurements revealed the sparse presence of larger structures in dilute acidic solutions of collagen. These could be removed by ultracentrifugation, and were shown not to affect the microrheology results reported herein.

For measurements probing collagen fibril assembly and gels, fibril formation was induced at room temperature (∼21°C) by adding 10× PBS buffer with excess phosphates to 5 mg/ml acid-soluble collagen to attain a final concentration of 0.5 mg/ml collagen, with salt concentrations of 273 mM NaCl, 5 mM KCl, 42 mM Na_2_HPO_4_ and 9 mM KH_2_PO_4_ (final pH = 6.9). To verify formation of fibrils under these conditions, samples were placed on copper grids, negatively stained with 2% uranyl acetate and imaged by transmission electron microscopy (TEM; Hitachi 8000). The kinetics of collagen fibril formation were monitored by changes in solution turbidity, by recording the increasing optical density at 347 nm (1700 UV-Vis Spectrometer, Shimadzu) as a function of time as fibrils assemble at room temperature [12].

For microrheology experiments, carboxy-terminated polystyrene microspheres (diameter 2.10 µm; Spherotech) were added to collagen solutions at a final concentration of ∼% w/v. In studies of collagen fibrils, the pH of the solution was then neutralized, so that fibrils self-assembled in the presence of the beads. Control experiments showed that this dilute concentration of beads did not affect the kinetics of fibril assembly, in contrast with observations at higher concentrations [38], [39]. ∼20 µl of prepared samples were pipetted into optical tweezers sample chambers, made of two microscope coverslides separated by a gasket cut from Parafilm.

When probing dynamical changes in local viscoelasticity during fibril assembly, the chamber was immediately mounted and aligned in the optical tweezers instrument. The time required for this mounting, alignment and stabilization of the instrument resulted in a delay time of approximately 20 minutes before microrheology measurements on fibril growth could be performed.

### Instrumentation

**Figure 1:**
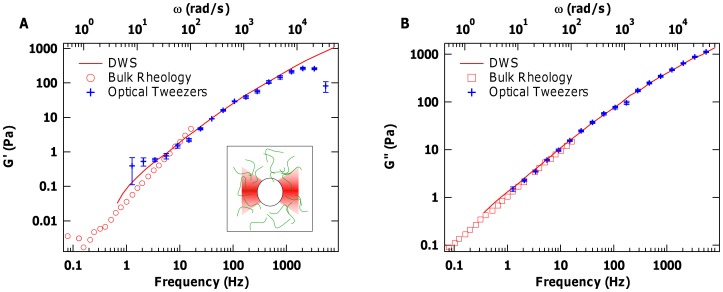
Validation of optical-tweezers-based microrheology results with measurements on PEO. (A) Elastic and (B) viscous moduli of 6.7 wt% PEO obtained using optical tweezers (crosses), compared with bulk rheology (open symbols) and microrheology via diffusing wave spectroscopy (DWS; solid line) from reference [46]. Crosses indicate the mean values over 5 independent measurements, and error bars represent the standard error of the mean. The inset shows a schematic of a probe particle confined by an optical trap inside a polymeric solution.

Details of the optical tweezers instrument used for microrheology assays have been previously reported [40], [41]. Briefly, a tightly focused laser beam (λ_0_ = 1064 nm, typical power ∼100 mW) is used to trap a microsphere (Figure 1(A) inset). After the laser beam traverses the sample chamber, a second objective lens collects the light, which is directed to a quadrant photodetector (QPD; QP154-Q-HVSD, Pacific Silicon Sensor), located to reimage displacements of the laser in the back focal plane of the second objective lens. The voltage output of the QPD is sampled at the desired bandwidth (here, 100 kHz) and sent to a computer for further analysis. The instrument is also equipped with a high-speed camera, which allows us to measure particle position at high bandwidth (>1 kHz). In these experiments, camera images were used to calibrate the QPD response for displacements of each probe particle. For measurements on collagen solutions, this Volts (QPD signal) to micrometer conversion factor was determined and used for each probe bead. However, for fibrillar collagen gels, we found reduced error in our results when we used the average value of the conversion factor extracted from our bead measurements in water (*N* = 28), combined with the subtraction of an average 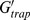
for these samples (see below).

During long-duration measurements monitoring collagen self-assembly, the laser beam was “blinked” off and on between measurements, so as to minimize radiation exposure and possible heating and reduce the likelihood of trapping growing fibrils in the optical trap, while attempting to maintain the same probe particle for multiple consecutive measurements.

### Principles of Microrheology and Analysis

As with other biopolymer materials, collagen systems exhibit viscoelastic behavior: they deform like a solid until the deformation is relaxed through liquid-like molecular reorganization [42]. We determine the complex shear modulus to obtain information about this time-dependent mechanical response of collagen systems to applied stress. In our microrheology experiments, the time-dependent motion of micron-sized tracer particles, obtained from their interactions with the trapping laser, is measured within collagen samples [43]–[45]. This optical tweezers implementation of microrheology enables the measurement of mechanical properties on the micrometer scale relevant to cells, in contrast to conventional rheology. It also probes small volumes (appropriate for assaying small amounts of sample) and allows read-out of the mechanical response at sub-millisecond timescales (bandwidths of >10 kHz), the latter a distinct advantage over video-based particle-tracking microrheology [23].

Viscoelastic properties can be obtained from the particle’s thermal fluctuations using the following method [43]. The power spectral density (PSD) of particle displacement is calculated from the extracted particle displacement as a function of time (QPD signal). Then, based on the fluctuation-dissipation theorem, the PSD is used to determine the dissipation contribution of the complex response function as a function of frequency (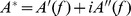):
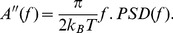
(1)


Here, *k_B_* is the Boltzmann constant and *T* is the sample temperature in Kelvin. Then, the real part of the response function is obtained by the Kramers-Kronig relation:
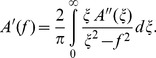
(2)


The real and imaginary parts of the complex shear modulus (*i.e.*, elastic and viscous shear moduli: 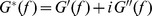) can then be determined by the complex response function.
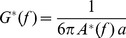
(3)where *a* is the bead radius. Within this manuscript, we present the magnitudes of the viscous modulus (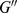), which is by definition negative. Unlike the viscous modulus, the resulting elastic modulus (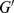) contains information on elasticity of both the trap and the medium, and has to be corrected as follows:



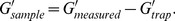
(4)When the procedure is applied to a Newtonian fluid such as water, since 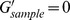, the measured 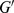 is due solely to the trap (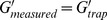). However, non-Newtonian systems (such as collagen solutions and gels) possess elasticity, whose magnitude must be determined from the measured values of 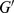. To extract 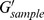 from the measured values, we used one of two different approaches, one for solutions of collagen molecules and the other for gels.

For collagen solutions, we use the value of 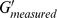 at the lowest frequency to represent 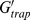 for each measurement. Because 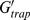 depends on bead size and laser power, this approach represents a good method for determining its value independently for each measurement. The validity of this approach is supported by the low-frequency plateau in 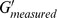 that scales with laser power (see Figure S1) and by the consistency of 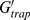 values found in this way with those of beads measured in water (see below). These features demonstrate that all of the collagen solutions probed in this study possess little elasticity at low frequencies.

For fibrillar collagen samples, however, the assumption of little elasticity at low frequencies is not generally valid. To correct for contributions from the trap, we therefore subtracted from each 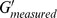 the average 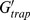 determined for these beads in water (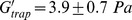 for the laser power of 100 mW used in these experiments). The variability in 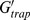 likely arises from the dispersion of bead sizes in our sample.

As a final step for both collagen systems, the resulting values for G* were averaged into logarithmically spaced frequency blocks in order to represent the data more clearly.

### Validating the Measurements of Complex Shear Moduli

In order to validate the values of shear moduli obtained from our optical tweezers microrheology measurements, we examined polyethylene oxide (PEO), a simple uncrosslinked polymer solution. 200 kDa PEO (Sigma Aldrich) was tested at concentration of 6.7 wt%, prepared as described [41], [46]. Figure 1 shows a comparison of the viscoelastic properties of PEO obtained from our measurements with previously published results using bulk rheology and another microrheology technique, diffusive wave spectroscopy (DWS) [46]. It is clear that our extracted values of 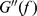 show excellent agreement over our entire measured frequency range, as do values for 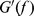
for most of the frequency range. At higher frequencies, the extracted values of 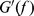
underestimate the true 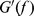, a well-known problem in microrheology that stems here from the finite bandwidth of our measurements compared with the infinite integration range in the Kramers-Kronig relation (equation (2)) [43].

For the solutions of collagen molecules, results were obtained for different laser powers (42, 100, and 150 mW), and results presented herein pool these measurements, showing that the reported results for 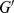 and 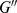 of collagen systems are independent of trap modulus within this range of 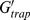
∼1–4 Pa. We also found that the complex shear modulus was not significantly affected by probe particle surface chemistry (carboxylated and aminated) or by collagen sample batch (probing other acid-soluble collagen from the same supplier).

## Results

### Viscoelastic Properties of Molecular Collagen Solutions

**Figure 2:**
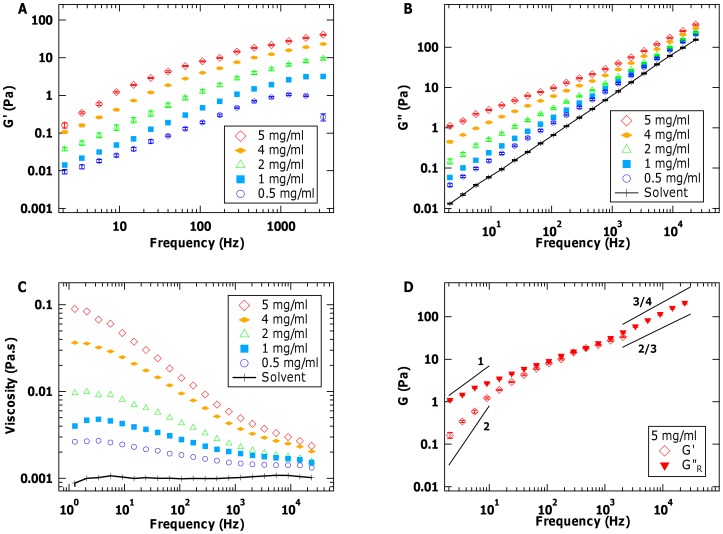
Concentration dependence of viscoelastic properties of solutions of molecular collagen. (A) Elastic and (B) viscous moduli increase with collagen concentration from 0.5 to 5 mg/ml (in 20 mM acetic acid). The viscous modulus of the solvent (20 mM acetic acid) is plotted in (B) for comparison. (C) The viscosity of collagen solutions (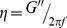 ) is also frequency-dependent and increases with collagen concentration. (D) The reduced viscous modulus, 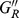 (filled triangles), is plotted along with elastic modulus for solutions of 5 mg/ml collagen. The solid lines at low frequency represent the expected Maxwell scaling of the moduli. At high frequency, the slopes indicate possible frequency-dependent scaling of moduli for flexible and semiflexible polymers. Values presented are averages over 10 independent measurements at each concentration of collagen, with error bars representing the standard error of the mean.

In order to characterize the interactions between collagen molecules, we investigated the viscoelastic response of collagen solutions as a function of concentration at acidic pH (where fibrils do not form). These samples were spatially homogeneous as seen by the minimal spread in values from independent measurements. Therefore, stated values of 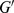 and 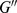 represent mean moduli, measured here from *N* = 10 different probe particles, with error bars representing the standard error of the mean values. As shown in Figure 2, we found that both elastic and viscous moduli increase with concentration, as does viscosity (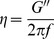). At 5 mg/ml, elasticity becomes comparable to viscous behavior at frequencies above ∼200 Hz.

At this highest concentration, molecular collagen solutions clearly possess frequency-dependent viscoelasticity. To quantify this, we analysed the power law scaling of 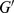 and of 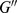 with frequency in different frequency ranges (Figure 2D). For 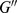, this scaling was determined from the reduced viscous modulus (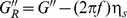, in which 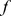 is frequency and 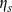 is solvent viscosity) [42]. At low frequencies (*f* <15 Hz), the log-log slope of 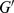 with frequency is ∼1.2, while that of 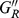 is ∼0.6. At intermediate frequencies (100 Hz<*f* <2 kHz), this scaling decreases to ∼0.5 for both 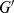
and 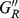. At the highest frequency range of our measurements (2 kHz<*f* <20 kHz), the scaling of 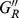 with frequency is ∼0.6.

### Viscoelastic Properties of Collagen Gels

**Figure 3:**
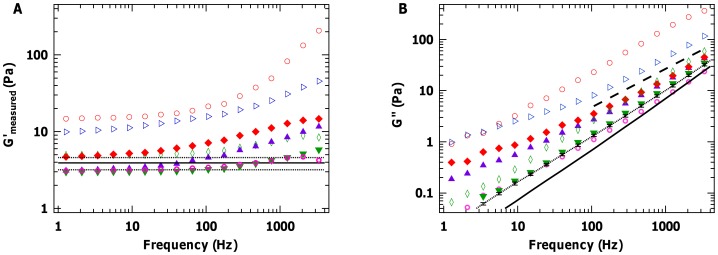
Heterogeneity of viscoelastic properties within collagen gels. Measured (A) elastic and (B) viscous moduli at different locations in collagen fibrillar gels (prepared from 0.5 mg/ml collagen at 21°C and pH = 6.9). Note the large variation in properties at different positions (within either the same gel or gels prepared under identical conditions). The solid and dashed lines in (A) represent the independently measured average elastic modulus of the trap and its standard deviation (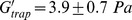) for the same laser power and bead size (*N* = 28). In (B), the dashed line at high frequency is plotted to illustrate power-law scaling of 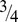. For comparison, the solid and dotted lines plot the measured viscous modulus of water and of 0.5 mg/ml collagen in acidic solution, respectively. Gel data shown here include the maximal and minimal moduli measured in all of our experiments.

Next, we used microrheology to probe local viscoelastic properties of self-assembled collagen systems. Figure 3 shows representative measured elastic and viscous moduli at different locations in collagen gels, formed from 0.5 mg/ml collagen at pH 6.9 at room temperature. As can be seen, the moduli (as measured using 2 µm-diameter probe particles) vary by more than an order of magnitude at different locations within the gel. In addition to the range of values of 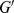 and 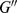, the frequency-dependent scaling of these moduli with frequency differs from one location to another (Figures 3 and S2). This is less pronounced for 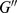 at high frequencies, where the curves appear to have a power-law scaling of close to 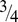. For 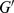, however, the variation of power-law scaling within these gels is particularly marked at high frequency, ranging from approximately 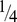 to 1.

It is important to note that the values plotted in Figure 3A for 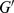 of these collagen gels are the measured values, which include a contribution of 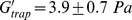 (indicated by the solid and dashed lines in Figure 3A) that should be subtracted from 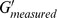 in order to extract the true elastic modulus of the fibrillar gel. The range of trap elastic moduli (most likely caused by the polydispersity of bead size) means that we cannot accurately determine 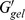 for each measurement. For regions of the gel with low elastic moduli, the uncertainty in trap stiffness leads to a significant range of possible slopes of log 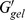 vs. log *f*, an issue that is not problematic for larger moduli (e.g. at higher frequencies) (Figure S2). Thus, we do not attempt to quantitatively analyse the low-frequency scaling of the elastic response of our collagen gels. Nonetheless, it remains clear that there is a strikingly broad range of both elastic and viscous moduli within the 0.5 mg/ml fibrillar collagen gel matrix (at *f* ≈ 40 Hz: 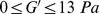, 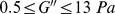; and at *f* ≈ 2000 Hz: 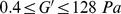, 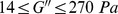; *N* = 40).

### Probing Dynamics of Collagen Self-assembly

**Figure 4:**
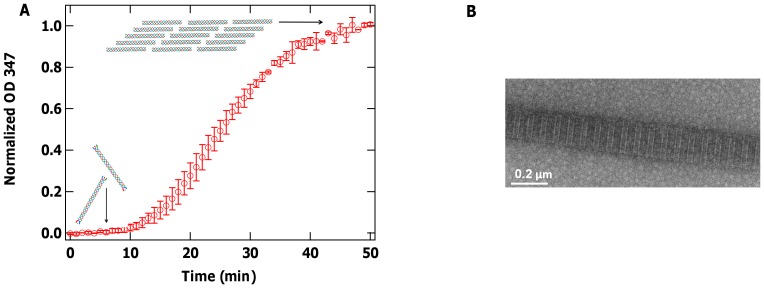
Kinetics of collagen fibril assembly. (A) Turbidity increases significantly as collagen self-assembles into fibrils. Experiments were conducted for a collagen concentration of 0.5 mg/ml (pH = 6.9 and 21°C). Open circles and error bars represent means and standard deviations from 3 replicate measurements. Inset schematics illustrate that the system begins as triple helical collagen molecules (more flexible than the rod-like schematic) and ends with well-ordered collagen fibrils (of which one small part is shown) that form a gel. (B) TEM image of a self-assembled collagen fibril formed under these conditions, showing the dark-light “D-banding” pattern expected for well-ordered fibrils.

We next aimed to investigate viscoelastic properties during the process of assembly from molecules into fibrils. Kinetics of collagen assembly were first measured via the increase in solution turbidity (Figure 4(A)). There are three distinct regions in this sigmoidal curve: a lag phase before the onset of increase in optical density during which fibrils nucleate, a growth phase during which they increase in size, and a plateau phase [8], [12], [24]. We chose to use a collagen concentration of 0.5 mg/ml for our microrheology measurements, enabling us to monitor mechanical changes at least from the early stages of fibril growth. Although lower collagen concentrations would further decrease the kinetics, allowing us to probe earlier stages of fibril assembly, the anticipated lower values of elastic and viscous moduli [47] would create further challenges for determination of the elastic modulus. Besides concentration, molecular composition and solution conditions such as pH, ionic strength and temperature affect kinetics of collagen self-assembly [8], [12], [14], [48]. In our experiments, we kept these constant between turbidity measurements and the following microrheology experiments. At the end of assembly, the products were imaged by TEM, and through the resulting size and periodic D-banding pattern, verified that well-ordered fibrils had been formed (Figure 4B) [8].

We now demonstrate that we are able to probe the development of broadly distributed viscoelastic properties over a wide range of frequencies during collagen fibril growth. In the measurements discussed here, we present elastic and viscous moduli determined from 47 independent measurements (different probe beads and locations) during the process of self-assembly.

**Figure 5:**
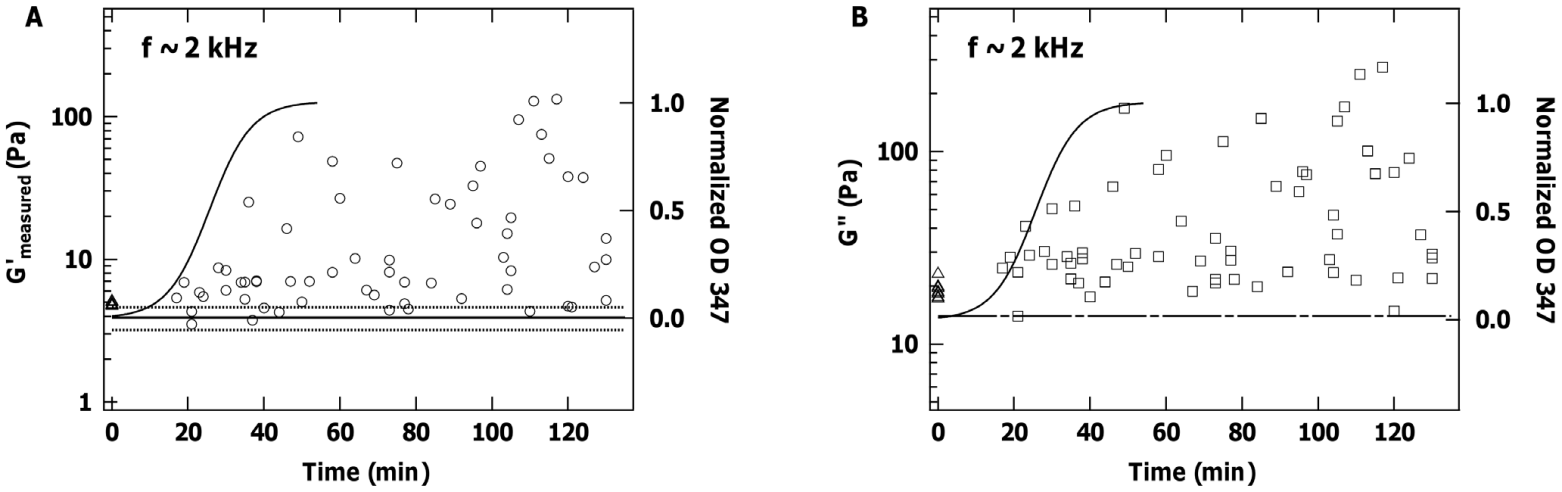
Heterogeneous microscale viscoelasticity is apparent even from the early growth phase of collagen fibril assembly. Measured (A) elastic and (B) viscous moduli at *f* = 2 kHz as a function of time during collagen self-assembly at 0.5 mg/ml (pH = 6.9, 21°C). Solid and dashed lines in (A) show 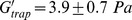
as described for Figure 3. The dot-dashed line in (B) plots the measured viscous modulus of water at *f* = 2 kHz. Triangles at zero time reproduce moduli measured at *f = *2 kHz for 0.5 mg/ml collagen in acidic conditions (Figure 2), where assembly cannot occur. For comparison, the sigmoidal development of turbidity (Figure 4) is superimposed on the plot (solid line; right axis), rescaled vertically between solvent response and the maximum measured value of each modulus.

In order to investigate development of mechanical heterogeneity, we measured the dispersion in elastic and viscous moduli as a function of time (after altering solution conditions to permit fibril assembly). To more clearly illustrate this development, in Figure 5 we plot the moduli at a high frequency (*f* = 2 kHz) versus time. This high frequency was chosen for the purposes of display for two reasons. First, the modulus of 0.5 mg/ml collagen in acidic conditions (shown for representative purposes as time = 0 in Figure 5) is greater than the trap modulus. Second, our measurements on gels at long times (>1 hour after formation; Figure 3) indicated that most elastic moduli of the sample were significantly larger than 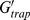 at this measurement frequency. Similar plots for both moduli at a lower frequency (*f* = 100 Hz) are shown in Figure S3.

The microrheology measurements during assembly indicate a dynamic system, in which mechanical heterogeneity is apparent even at the early stages of the growth phase. As time passes, the variation in moduli increases, reaching a plateau after ∼50 minutes. For instance, according to Figure 5, at time ∼20 minutes, 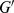
values at different locations were observed to range between that of the trap (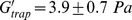) and ∼7 Pa; whereas at longer times (e.g. ∼50 minutes), they spanned a range between 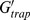 and ∼70 Pa. Similarly, development of heterogeneity was observed for 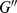
(at t∼20 min: 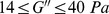, while at t∼50 min: 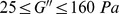).

In Figure 5 triangles at zero time reproduce moduli (at *f* = 2 kHz) of 0.5 mg/ml collagen in acidic conditions, where fibril formation cannot proceed (Figure 2). Although ten different probe particles (different locations) were included for this zero-time condition, as stated above their lack of significant dispersion demonstrates that the collagen solutions are homogenous prior to the initiation of fibril formation.

## Discussion

### Viscoelastic Properties of Molecular Collagen Solutions

Our results on solutions of molecular collagen show that 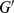 and 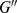 increase with concentration, expected behaviour as the presence of more polymers in solution and interactions between them strengthen the viscoelasticity of the system.

The magnitudes of 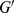 are smaller than 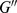 at all but the highest concentration studied, showing that collagen molecules in acidic solution represent a system dominated by viscous interactions. The low elasticity likely arises from limited entanglement between collagen molecules due to their short contour length (300 nm) [43], [49], and from limited intermolecular attraction, arising from their overall positive charge at this acidic pH and the shielding of electrostatic interactions beyond intermolecular separations of ∼13 nm (the Debye length for the 0.6 mM ionic strength of 20 mM acetic acid).

There is, nonetheless, clear evidence that interactions between chains, whether through entanglement, local electrostatic or biochemical interactions, do occur, as the shape of the frequency-dependent moduli change with increasing collagen concentration. At the highest concentration measured here (5 mg/ml), the elastic modulus becomes comparable in magnitude to the viscous modulus at frequencies above ∼100 Hz, demonstrating the role that intermolecular interactions play in conferring elasticity to the system on the few millisecond timescale.

At sufficiently low frequencies, the response of these collagen solutions is governed by their slowest relaxation process and should approach the Maxwell limit: 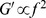 and 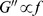 (which are shown as solid lines at low frequencies in Figure 2D). For dilute semiflexible chains in solution, the slowest relaxation process is governed by entropic orientational reorganization (well described by rotational diffusion of rigid rods) [50]. For higher concentrations, this relaxation process involves disruption of interactions between chains, which can include hydrophobic or other noncovalent interactions as well as physical entanglement [51]. At the lowest frequencies of our measurements, we find frequency-dependent scaling of 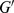 that increases with concentration (from ∼0.7 to ∼1.2; Figure 2A), while the exponent best describing the low-frequency relationship between 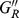 and frequency decreases with concentration (from ∼0.9 to ∼0.6; the trend can be seen in 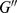 versus frequency in Figure 2B). Thus, down to frequencies of 1 Hz, collagen solutions in this concentration range do not exhibit Maxwell scaling. Future studies extending the measurement times could investigate whether Maxwellian behavior is achieved and at what timescale. Our previous findings that a substantial increase in ionic strength lowers both the elastic and viscous moduli of 5 mg/ml collagen in this low-frequency regime [41], moving their frequency-dependent scaling closer to the Maxwell limit, suggests an alternative means to achieve this limit.

For the highest collagen concentration in our work, 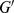 and 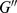 become comparable in magnitude and slope in the intermediate frequency range (∼100 Hz –2 kHz). The softening of the slope in this range, rather than a true plateau, indicates that the concentration is high enough that chains become interacting, and that relaxation via chain curvature and tension both contribute in this frequency interval [52].

At sufficiently high frequencies, the response of the system should be that of isolated chains in solution, at which point the power-law scaling of both moduli approaches the same limit in an isotropic system [43], [52], [53]. For the highest concentration probed, the slopes of 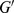 and 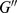 are similar in the mid-frequency range (100 Hz<*f* <2 kHz) of our measurements (Figure 2D). Because only the viscous modulus 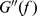 can be correctly determined at high frequency (*f* >2 kHz) from these optical tweezers measurements, we examine its scaling in this short-time regime. At the highest concentration studied here, the high-frequency (2–20 kHz) slope of log(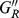) vs. log(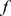) is approximately 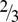 (Figure 2D), a value commensurate with predicted scaling for Zimm polymers [42]. Numerous lines of evidence suggest, however, that we are not measuring at high enough frequency to probe the dynamics of individual chains.

First, because estimates of collagen’s persistence length (*L_p_*) range by an order of magnitude, from ∼15–160 nm [17], [18], [54]–[56], giving a ratio of 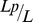 ranging from 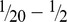 when compared with its contour length of *L* = 300 nm, it may fall in the “crossover region” between rigid rods and random coils in which *L* is not much larger than *L_p_* [50]. These previous measurements do not resolve whether collagen is better considered as semiflexible or freely jointed, a question of flexibility which the high-frequency scaling of the complex shear modulus could address. For semiflexible chains, the high-frequency scaling of both moduli should approach 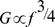 [52], [57], while the Zimm model for random coils predicts a 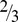 scaling, as we observe for 5 mg/ml collagen at our highest measured frequencies. (Semiflexible chains may also exhibit 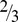 scaling at intermediate frequencies, through the relaxation of long-wavelength (>*L*_p_) modes [52].) Because this observed scaling does not hold at lower concentrations (see below), it is unlikely we are capturing the short-range relaxation of isolated chains in this frequency range. To resolve this, measurements at higher frequency (shorter relaxation timescales) could be performed to assess the true high-frequency scaling relationships attributable to individual chain dynamics. Computer simulations probing the viscoelastic response of a single collagen triple helix found that intramolecular relaxation occurs on the timescale of a nanosecond [19], orders of magnitude faster than accessible with optical-tweezers-based microrheology.

Second, we find that the high-frequency scaling exponent depends on collagen concentration. As concentration decreases, the exponent increases, reaching ∼0.9 for 0.5 mg/ml. This is significantly larger than the conventional 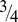 relationship expected for semiflexible chains and is in closer agreement with the 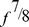 scaling recently found for certain high-frequency relaxation modes in these systems [53]. 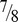 scaling occurs for longitudinal relaxation (parallel to the local polymer chain), and these modes are likely to dominate over transverse relaxation (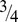 scaling) for anisotropic systems or strained polymers [53], [58]. In the isotropic collagen solutions of this part of our study, 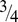 scaling is expected for semiflexible chains, and our results thus suggest that even for the most dilute samples studied here, interchain interactions contribute to the viscoelasticity at ∼10 kHz (100 µsec timescales).

**Figure 6:**
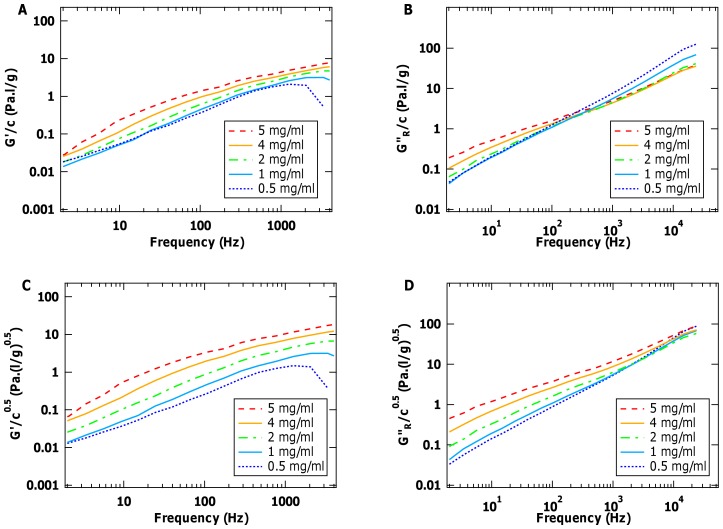
Concentration dependence of the viscoelastic properties of collagen molecules in solution. (A) Elastic and (B) reduced viscous moduli (Figure 2) divided by sample concentration, *c*. While the lowest concentration of 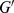 values appear to scale linearly with concentration, this is not the case for 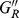. Instead, dividing by the square root of concentration appears better to rescale the reduced viscous modulus at high frequency (D), although not the elastic modulus (C).

Third, if viscoelasticity is due only to the dynamics of isolated chains, we expect the moduli to scale linearly with concentration, as more (isolated) molecules simply increase the amount of elasticity and dissipation experienced by the probe particle. To investigate this concentration dependence, in Figure 6 we rescale the moduli 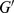
and 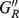 by dividing by collagen concentration, *c*. In the intermediate frequency range, the linear rescaling with *c* provides curves of 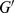 that appear to converge at the lowest concentrations. However, this does not happen with 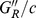, where the relatively larger rescaled values for the lowest concentrations imply that the reduced viscous modulus scales sublinearly with concentration. Solutions of polymers are expected to undergo concentration-dependent changes in the scaling of specific viscosity with frequency. In the dilute limit, *η_sp_* scales as *c* ^1^, which then turns over to *η_sp_* 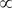 *c* ^0.5^ as concentration is increased, before scaling as *c* ^1.5^ for highly entangled systems [59], [60]. Correspondingly, we tested whether the concentration range studied here might better be represented by a *c* ^0.5^ dependence of 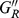. As seen in Figure 6D, rescaling 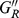 by dividing by *c* ^0.5^ produces curves that more closely converge in the high-frequency limit. This third piece of evidence further supports the likelihood that our samples are not in the dilute limit, even at 1 mg/ml.

The finding that collagen concentration should be below 1 mg/ml to be in the dilute regime contrasts with an earlier estimate of an overlap concentration of ∼2.5 mg/ml for collagen in acidic solution [61]. (The overlap concentration is the concentration at which molecules in a solution start to physically contact, which therefore changes the scaling of viscoelastic properties of the solution [42].) Analysis of our results for the power-law scaling of specific viscosity versus concentration also showed the transition to the dilute regime to lie close to 1 mg/ml (data not shown). At this concentration, the average distance between isotropically distributed chains in solution would be 73 nm (using a molecular weight of 300 kDa for collagen). This separation is less than collagen’s contour length of 300 nm, while it is larger than its estimated radius of gyration were it a chain with the shortest estimated persistence length, *L_p_* = 15 nm. Thus, an overlap concentration below 1 mg/ml is physically reasonable. To achieve insight into the scaling laws of isolated collagens, and to confirm that they are semi-flexible, requires either extending the measurements to higher frequency and/or to lower concentrations.

The concentrations we have investigated are lower and the frequencies higher than most previous studies of collagen viscoelasticity [54], [56], [61], [62], and, for the first time, apply the technique of microrheology to examine acidic solutions of collagen. There is wide variation among the few published values for 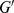 and 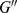 for this low range of concentrations of collagen in acidic conditions. The magnitudes we find are very similar to the earliest published rheology studies by Nestler *et al.* across their entire studied frequency range (150–8000 Hz), and, consistent with their findings across a range of collagen concentrations *c* <1 mg/ml, that 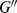 is larger than 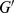 [56]. A more recent study found 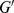 and 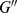 to be considerably larger (e.g. 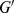
>10 Pa vs. 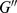 ∼3 Pa for 5 mg/ml at 10 Hz) and have commensurately lower crossover frequencies, at which 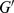 and 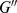 become comparable [62]. We are confident in our results for the following reasons. Although our correction for 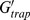 may result in a slight underestimation of 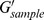 at low frequencies, our values of 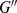 are not influenced by this correction factor. By probing response using beads with different surface chemistry (Materials and Methods) and by significantly increasing the ionic strength of the acidic solution [41], we found no changes in the magnitudes of the moduli, demonstrating that electrostatic depletion does not significantly contribute to our results. (Entropic depletion, due to exclusion of certain filament orientation near the bead’s surface, could be investigated using different sizes of probe particles [63].) We have verified our sample concentrations by ELISA, and have determined via ultracentrifugation and dynamic light scattering that the viscoelasticity we report is due to non-aggregated samples. We have also performed CD spectroscopy on the collagen samples to verify that it has triple helical structure, and the well-ordered fibrils that result from self-assembly (Figure 4B) further support its correct molecular structure.

### Viscoelastic Properties of Fibrillar Collagen Gels

From isolated molecules in acidic solution, we then probed the viscoelasticity of collagen gels resulting from self-assembly into fibrils at neutral pH. First, we discuss the microrheological properties of these gels, and in the following section, discuss how our measurements on the development of their properties relate to models of their formation mechanism.

Microscale inhomogeneity within collagen gels is clearly evident in our microrheology measurements (Figures 3, 5, S6 and Movie S1). The magnitudes of both moduli range from approximately that of the solvent (plus optical trap for 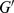) to over 100 Pa (at *f* = 2 kHz). Independent measurements on a collagen gel formed from 1.0 mg/ml collagen at 31°C, probed at room temperature, suggest that it exhibits a comparable range of microscale complex shear moduli (Figure S4). The moduli we obtain span the values from bulk rheology measurements on collagen gels of comparable concentration at the appropriate comparison frequency [25], [27], [28], [64]. For the more gel-like regions of the sample, 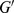 dominates over 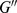 at low frequencies (confirming the elastic nature of these gels), while at high frequencies, 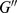 is larger.

Variation of microscale viscoelastic properties within formed collagen gels, ranging from solvent-like to elastic response, has been noted previously by others [23], [26], [29]. These previous microrheology studies on collagen gels used pepsin-solubilized collagen, which lacks its telopeptide ends and has a reduced ability to form fibrils [65]. Our results, on acid-solubilized collagen, demonstrate that this microscale variation in response applies also to fibrillar gels formed from full-length collagen (*i.e.*, including the telopeptide ends). In addition, the current work significantly extends the frequency range of response probed in these systems, which had previously been studied only up to ∼10 Hz with passive microrheology [23] or at a specific frequency of ∼5 Hz [29] or 100 Hz [26] with active microrheology. As a result, our results show not only the range of collagen gel moduli experienced by a microsphere at one frequency but also the variation in power-law scaling both of 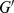 and of 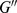 at different regions within a gel (e.g. Figures 3 and S2). Such variation in scaling was not detected in the lower frequency measurements of [23], where 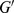 appeared to be frequency-independent.

Measurements in regions of higher elasticity demonstrate a clear plateau of 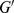 versus frequency which extends to frequencies at least as low as 1 Hz (Figures 3 and S2). Because in our measurements no crosslinking agents are used (similar to most other studies of collagen gels [64]), the interactions between fibrils generating gel-like behavior must be transient, resulting from entanglement and/or biochemical and electrostatic interactions rather than from permanent covalent crosslinks between segments of different fibrils. At sufficiently long times (low frequencies), the plateau in 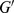 should turn over into the terminal regime [57].

At higher frequencies, we observe a broad range of frequency-dependent power-law scaling of 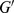 (e.g. Figure S2), which at the highest frequencies in our measurements ranges from ∼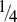 for regions of lower elasticity to ∼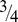 for regions of higher elasticity. For 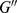, the high-frequency scaling ranges from ∼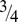 to ∼linear, as seen in Figure 3B, where the dashed line represents a scaling of 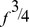. The 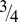 scaling is expected for semiflexible filaments when individual filament relaxation dominates the response. It is important to note that in contrast with the results discussed in the previous subsection, here, the semiflexible filaments would be fibrillar collagen (diameters ∼200 nm; Figure 4B) rather than single collagen triple helices (diameter ∼2 nm). While fibrillar type I collagen can reach tens of µm in contour length, for gels, the relevant contour length, particularly at these high frequencies, is the distance between entanglements rather than the full contour length. For example, gels made from 1 mg/ml collagen have an average filament length between nodes of the network of 2.0 µm [66]. A similar frequency-dependent scaling exponent of 0.70 for 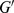 and 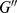 was found in the growth of collagen fibrillar gels analysed using percolation theory [39].

Work by Piechocka and colleagues has found that collagen gels undergo non-affine deformation, precluding detailed quantitative analysis [27]. Nonetheless, a question that arises is what contributes to the measured viscoelastic response for the wide range of moduli measured here. As described above, the “struts” in the collagen gel are fibrils, which individually have elastic moduli orders of magnitude higher than the shear elastic moduli of our gels [22]. Our lower measured values result from the very low strains imposed by this passive microrheology technique [67]. Between these struts are pores whose typical size is larger than our 2 µm probe particles [25], [28], although there is a broad distribution of sizes that extends down to this particle size [28]. Using probe microspheres of a similar size to ours in active microrheology experiments, Latinovic *et al.* specifically probed sparse regions of a 2 mg/ml collagen gel and found no detectable elasticity at 100 Hz [26]. Their values for 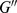 at 100 Hz in these regions were moderately larger than values measured in their control solvent, although similar within error. They interpreted this to mean that the sparse regions of their gel were completely depleted of molecular collagen, *i.e.*, that fibrillar gel formation had gone to completion. The interpretation of our results, from passive microrheology measurements on 0.5 mg/ml gels over a broad frequency range, is less clear cut. As seen in Figure 3, which shows representative frequency-dependent moduli (including the weakest modulus measured in our collagen gels), all of the 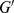 curves show some frequency dependence, especially at high frequency. Were the response due only to the optical trap, 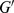 would not increase with frequency. For 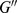 as well, the measurements that most closely approach solvent response (solid line in Figure 3B) do so at high frequency but deviate significantly at low frequency, tending toward the values measured for 0.5 mg/ml collagen molecules in acidic conditions (dotted line in Figure 3B). (The complex shear modulus of molecular collagen solutions at neutral pH and high ionic strength likely differs from that in acidic conditions, but we are unable to measure their response at ambient temperature due to their self-assembly into fibrils [54].) Because we determine 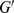 and 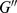 over a broad frequency range, the changes in curvatures of these moduli (and not just their values at one given frequency, which have a considerable uncertainty for small 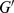) indicate that the local response measured in this gel does not range simply from that of fully formed fibrils down to fully depleted solvent. The presence of elasticity and viscosity above that of the solvent in our measurements could potentially result from occasional interactions with nearby fibrils, or alternatively from unincorporated molecular collagen in void regions of the gel. The latter seems an unlikely explanation, since collagen has been found to be almost completely incorporated into gels at this concentration and temperature [12]. The significantly larger number of probed locations in our measurements (*N* = 47 independent measurements on gels) compared with the active microrheology measurements of Latinovic *et al.* suggests that our probe particles do not often encounter a region of pure solvent. Experiments utilizing smaller probe particles and/or particles specifically bound to collagen fibrils would move toward measurements more comparable to the size of focal adhesions or to the sensing of fibril-specific viscoelastic response [1], [22], [67].

### Kinetics and Dynamics of Collagen Self-assembly into Fibrillar Gels

To correlate the development of viscoelasticity with the self-assembly of collagen into higher-order structures, we measured both the complex shear moduli and turbidity as a function of time. Turbidity is sensitive to the size of particles in solution, measuring the attenuation of transmitted light via scattering in a sample and is the conventional means by which the kinetics of collagen self-assembly into fibrils is followed [8], [12]. Our turbidity measurements were performed on sample volumes of 100 µl (pathlength 1 cm) and thus were insensitive to µm-scaled differences in solution composition. In contrast, the complex shear moduli from microrheology measurements reflect the environment around each µm-sized probe particle.

Our time-dependent measurements show that heterogeneity exists at timescales at least as early as 20 minutes after the initiation of assembly, and that it appears to develop further with time (Figures 5, S5). Active microrheology measurements similarly have found an increase in 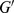 and 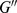 with time, as collagen fibrils grow and the gel develops [26]. The range of moduli that remains long after assembly has completed (based on the plateau in the turbidity curve) is evidence of the variety of local environments within this gel (as described in the previous sections).

We found it challenging to monitor the development of viscoelasticity at any given location as a function of time. Interestingly, during fibril assembly, beads were frequently observed to be forced out of the optical trap, presumably by growing fibrils. From the trap stiffness and bead size of our measurements, we conservatively estimate that this corresponds to a force of *F* >50 pN. While this mechanism remains to be investigated, self-assembly of other proteins, such as actin and tubulin, into polar filaments are biologically critical force-generating processes [68], [69]. Further challenges to following the local development of viscoelasticity resulted from our use of a “blinking” optical trap during these long duration measurements (see Materials and Methods), resulting in loss of beads due to diffusion or fibril growth during the times when the laser was off. Occasionally, we were able to monitor the local environment over extended periods of time, in one example observing a significant increase in 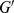 and 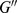 well beyond an hour and in another finding no evolution of these values with time (Figure S4). In the former case, our measurements suggest that elasticity and viscosity may continue to evolve locally within a gel, even when these properties have reached their maximum values elsewhere in the sample and turbidity measurements indicate that the gel has fully assembled.

In order to interpret the correlations between our microrheology results and turbidity measurements, we follow the percolation model discussed in [39]. Forgacs *et al.* have shown that collagen fibrillar gel assembly is initiated by random nucleation of filaments that grow in the form of clusters [39]. These randomly distributed clusters grow both longitudinally and laterally during the growth phase [24], which in our measurements results in a rapid increase in turbidity. Once these clusters grow sufficiently large, they become interconnected. In microrheology measurements, while the complex shear modulus is a function of the local environment about a microsphere, its magnitude, particularly at lower frequencies, is strongly affected by interactions within a larger network. Thus, as time evolves, we expect to see a transition from the low viscoelasticity of the isolated molecules in solution, through a variable range of properties that reflects whether we are measuring close to or far from isolated clusters, to finally locally probing the properties of the interconnected matrix. In Figures 5 and S3, the normalized turbidity curve is superimposed on the time-dependent measurements of complex shear moduli. As can be seen, the turbidity appears to form an envelope under which the shear moduli develop. By ∼ 1 hour after initiation of fibril formation, both turbidity and the maximum measured elastic and viscous moduli appear to plateau, although as mentioned above, the moduli may continue to develop locally. For all times measured, there remains great dispersion among values of both 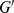 and 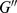, reinforcing the concept of a mechanically heterogeneous gel at the microscale. Future microrheology measurements during the nucleation and onset of collagen fibril growth phases would help to elucidate whether isolated regions of higher viscoelasticity are present in these early stages of self-assembly, as would be predicted for sparsely distributed clusters within the percolation model.

Further measurements would also be needed to determine whether isolated collagen gels can dynamically reorganize at the microscale (e.g. Figure S4), as is seen in response to applied stress [64], and to assess more quantitatively the percolation mechanism in the context of turbidity and microrheology measurements. Although correlations between structure and mechanics during the formation of collagen gels have been probed by combining rheology and imaging [24]–[26], [39], these have not assayed the associated development of turbidity of the system. A comparison with turbidity is useful given its prevalence in measuring the effects of molecular conditions such as collagen sequence and solution pH on fibril formation [8], [12], [14], [48].

### Conclusions

In this work, we investigated micromechanical properties of different hierarchical structures of collagen. In solutions of triple-helical collagen molecules, we observed concentration-dependent viscoelasticity, finding that elasticity at the highest concentrations of our study is due to collagen molecules interacting on millisecond timescales, in solution conditions that do not lead to self-assembly. Following a change in solution conditions to promote collagen self-assembly, we were able to monitor development of higher-order structure during this process via mechanical changes at the microscale. These changes exhibited significant spatial heterogeneity in response over orders of magnitude in frequency. The marked difference in both magnitude and type of power-law behavior of the complex shear modulus at different regions of the final fibrillar gel indicates that the types of interactions responsible for elasticity in these collagen matrices vary significantly on the microscale. This heterogeneity could be due to a variation in the length of collagen fibrils between entanglements, to the relative sizes of probe particles versus pore sizes, and/or to dispersion in the diameter of the fibrils [24], [28], [66]. We also observed that the growth of collagen fibrils generated sufficient force to dislodge probe microspheres from the optical trap. The force generation and gel properties of collagen fibrils bear similarities to biological filaments such as actin or microtubules, although the range of possible fibril diameters for collagen represents a distinct and potentially important control parameter for collagen’s mechanical interaction with cells and guiding of cellular fate.

## Supporting Information

Figure S1
**The measured elastic modulus for collagen solutions is dominated at low frequency by the trap modulus.** The elastic modulus of a solution of 5 mg/ml collagen is plotted for the same probe particle measured at two different laser powers (100 and 150 mW, filled square and empty circles, corresponding to smaller and larger 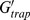, respectively).(TIF)

Figure S2
**Uncertainties in trap modulus significantly affect observed power-law scaling.** Here, two elastic moduli measured for collagen gels, 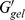, are obtained by subtracting 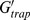 from the measured values of 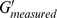 (same symbols as used in Figure 3). Symbols indicate the values obtained by subtracting the mean trap modulus from 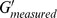, while the shaded regions for diamonds and error bars for circles represent the values obtained when considering the standard deviation of the elastic modulus of the trap (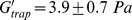). The power-law scaling in regions of the gel with 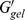
>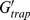 is affected very little by this uncertainty (upper curve), while the power-law scaling in regions of low elastic modulus is highly dependent on the specific value of 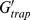 used (lower curve, particularly at low frequency where the slopes from the upper and lower bounds of 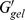 are clearly different).(TIF)

Figure S3 **Heterogeneous microscale viscoelasticity is apparent even from the early growth phase of collagen fibril assembly.** These data result from the same measurements as Figure 5 but here the values of the moduli at *f* = 100 Hz are plotted.
(TIF)

Figure S4 **Heterogeneity of viscoelastic properties within a collagen gel prepared under different conditions.** Measured (A) elastic and (B) viscous moduli at different locations in a collagen fibrillar matrix formed from 1 mg/ml collagen and prepared at 30°C and pH = 6.9. The solid line and dotted lines plot the measured viscous moduli of water and of 1 mg/ml collagen in acidic solution, respectively. Overall, the heterogeneity of viscoelastic properties within the gel does not change substantially with this different collagen concentration and formation temperature. Measurements were performed at room temperature.
(TIF)

Figure S5 **Time-dependent evolution of local viscoelasticity as collagen self-assembly proceeds.** Measured (A) elastic and (B) viscous moduli in an assembling collagen fibrillar gel, reproduced from Figure 5. In rare circumstances, we were able to retain a given probe particle for measurements at multiple timepoints. These results are indicated by filled symbols, and are connected by lines to guide the eye. A significant evolution in viscoelastic properties is seen at one location (solid line), while in another location, no significant change is observed (dotted line). Further experiments would help to elucidate whether local moduli can both increase and decrease with time, or whether for example the transient decrease in moduli around 100 minutes is due to drift of the sample chamber.
(TIF)

Figure S6 **Heterogeneity of properties within a collagen gel.** Plots here show the XY trajectories from Movie S1 of the (A) left bead and (B) right bead over ∼9 seconds. The motion of the left bead is more constrained (higher local elastic modulus), while the right bead exhibits substantial anisotropy of its motion.
(TIF)

Movie S1 **Heterogeneity of properties within a collagen gel.** The movie shows the movement of two (non-trapped) beads diffusing in a collagen gel sample prepared at 0.5 mg/ml collagen concentration and at room temperature.
(GIF)
