# Correction: Microrheological Characterization of Collagen Systems: From Molecular Solutions to Fibrillar Gels

**DOI:** 10.1371/journal.pone.0102938

**Published:** 2014-07-17

**Authors:** The PLOS ONE Staff

In the Materials and Methods, the final concentration listed for the carboxy-terminated polystyrene microspheres added to collagen solutions is incorrect. The correct concentration should appear as ∼
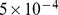
% w/v.
