# Supplementary figures and images for: Microrheological Characterization of Collagen Systems: From Molecular Solutions to Fibrillar Gels
Source: PLoS One. 2013 Aug 2;8(8):e70590. doi: 10.1371/journal.pone.0070590 (PMC3732230; doi:10.1371/journal.pone.0070590)

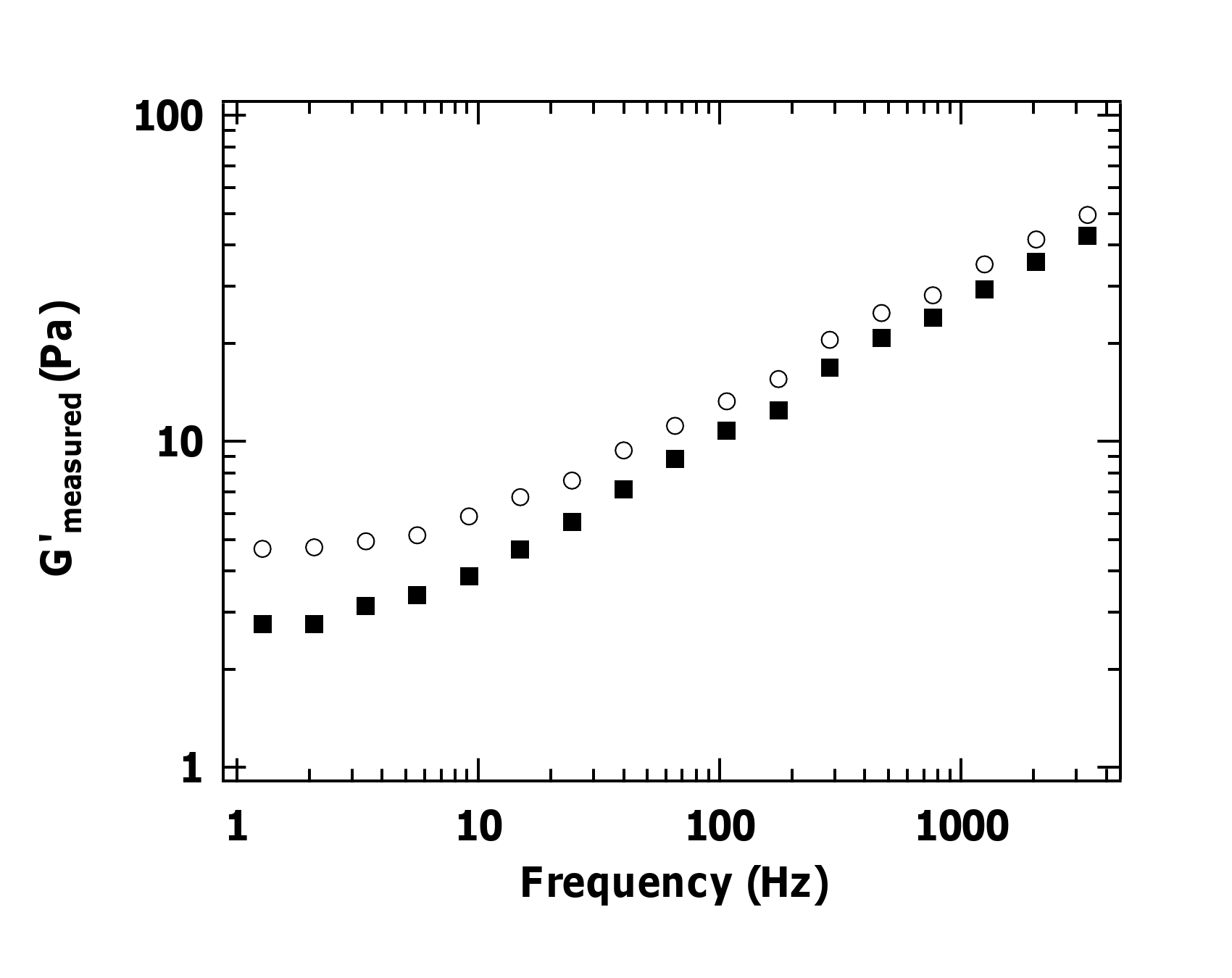

Supplement: Figure S1 — The measured elastic modulus for collagen solutions is dominated at low frequency by the trap modulus. The elastic modulus of a solution of 5 mg/ml collagen is plotted for the same probe particle measured at two different laser powers (100 and 150 mW, filled square and empty circles, corresponding to smaller and larger , respectively). (TIF) [file pone.0070590.s001.tif]

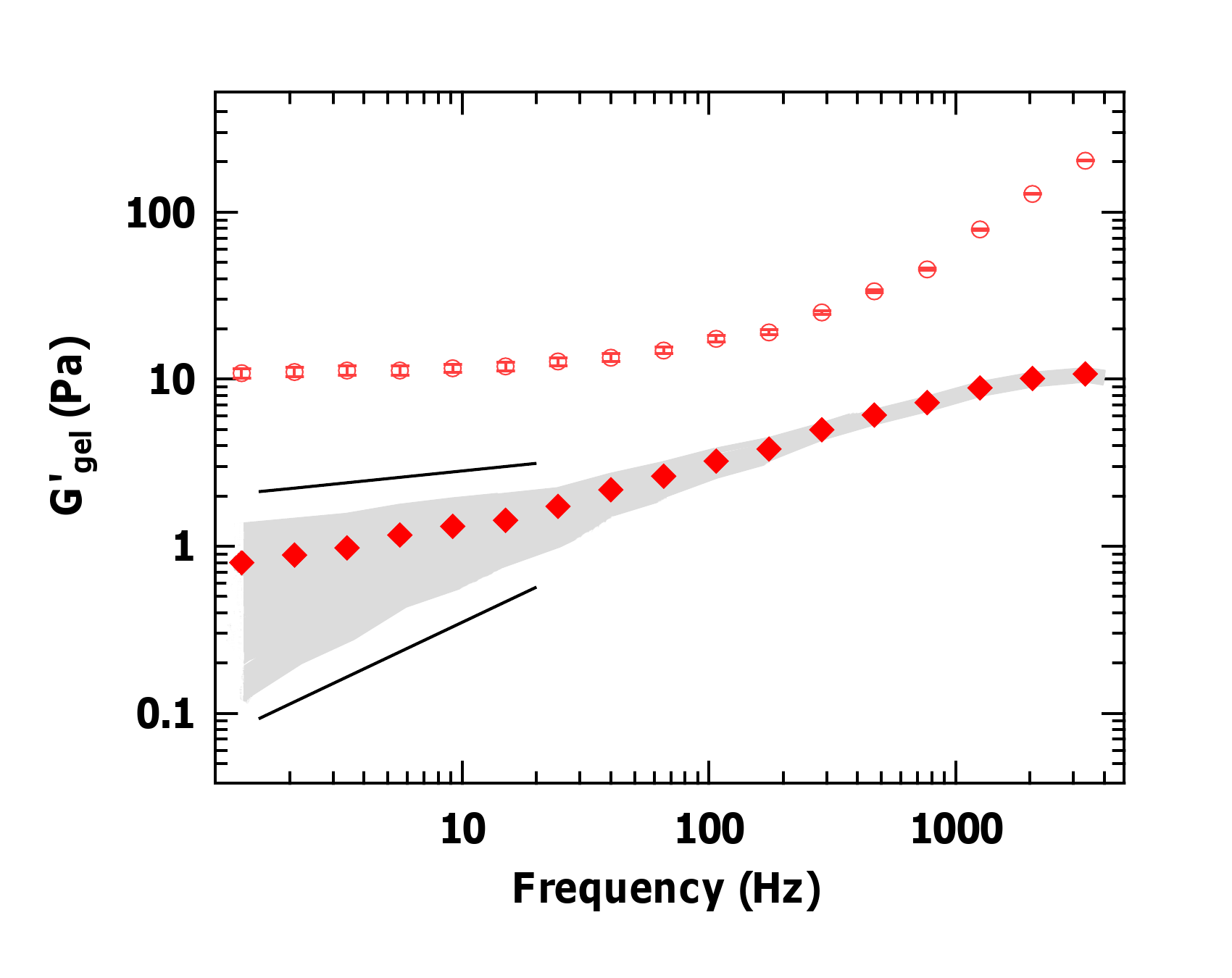

Supplement: Figure S2 — Uncertainties in trap modulus significantly affect observed power-law scaling. Here, two elastic moduli measured for collagen gels, , are obtained by subtracting from the measured values of (same symbols as used in Figure 3). Symbols indicate the values obtained by subtracting the mean trap modulus from , while the shaded regions for diamonds and error bars for circles represent the values obtained when considering the standard deviation of the elastic modulus of the trap (). The power-law scaling in regions of the gel with > is affected very little by this uncertainty (upper curve), while the power-law scaling in regions of low elastic modulus is highly dependent on the specific value of used (lower curve, particularly at low frequency where the slopes from the upper and lower bounds of are clearly different). (TIF) [file pone.0070590.s002.tif]

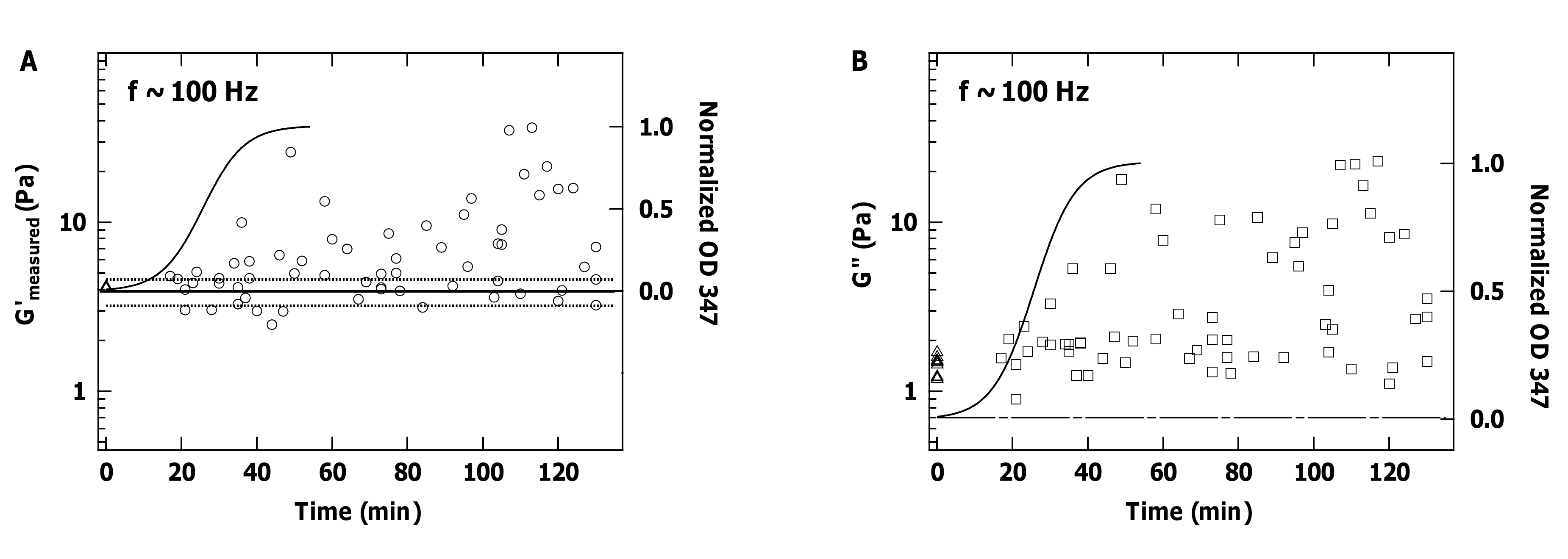

Supplement: Figure S3 — Heterogeneous microscale viscoelasticity is apparent even from the early growth phase of collagen fibril assembly. These data result from the same measurements as Figure 5 but here the values of the moduli at f = 100 Hz are plotted. (TIF) [file pone.0070590.s003.tif]

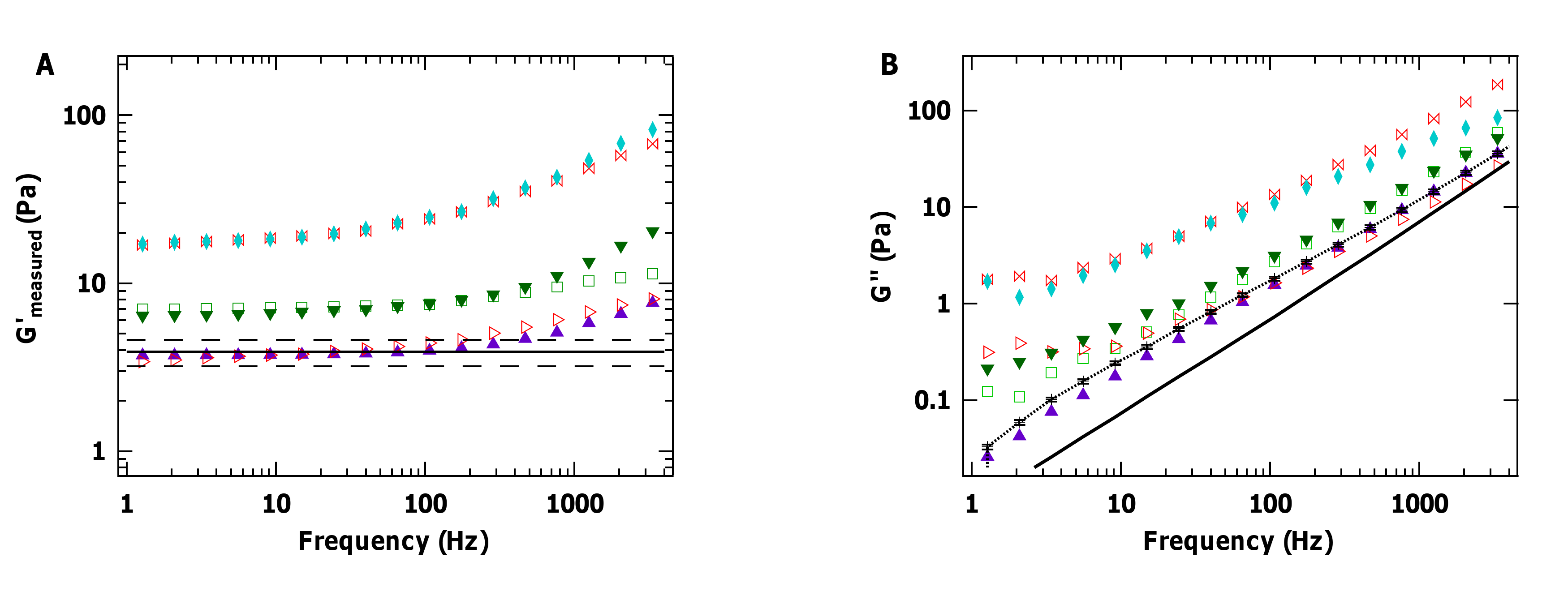

Supplement: Figure S4 — Heterogeneity of viscoelastic properties within a collagen gel prepared under different conditions. Measured (A) elastic and (B) viscous moduli at different locations in a collagen fibrillar matrix formed from 1 mg/ml collagen and prepared at 30°C and pH = 6.9. The solid line and dotted lines plot the measured viscous moduli of water and of 1 mg/ml collagen in acidic solution, respectively. Overall, the heterogeneity of viscoelastic properties within the gel does not change substantially with this different collagen concentration and formation temperature. Measurements were performed at room temperature. (TIF) [file pone.0070590.s004.tif]

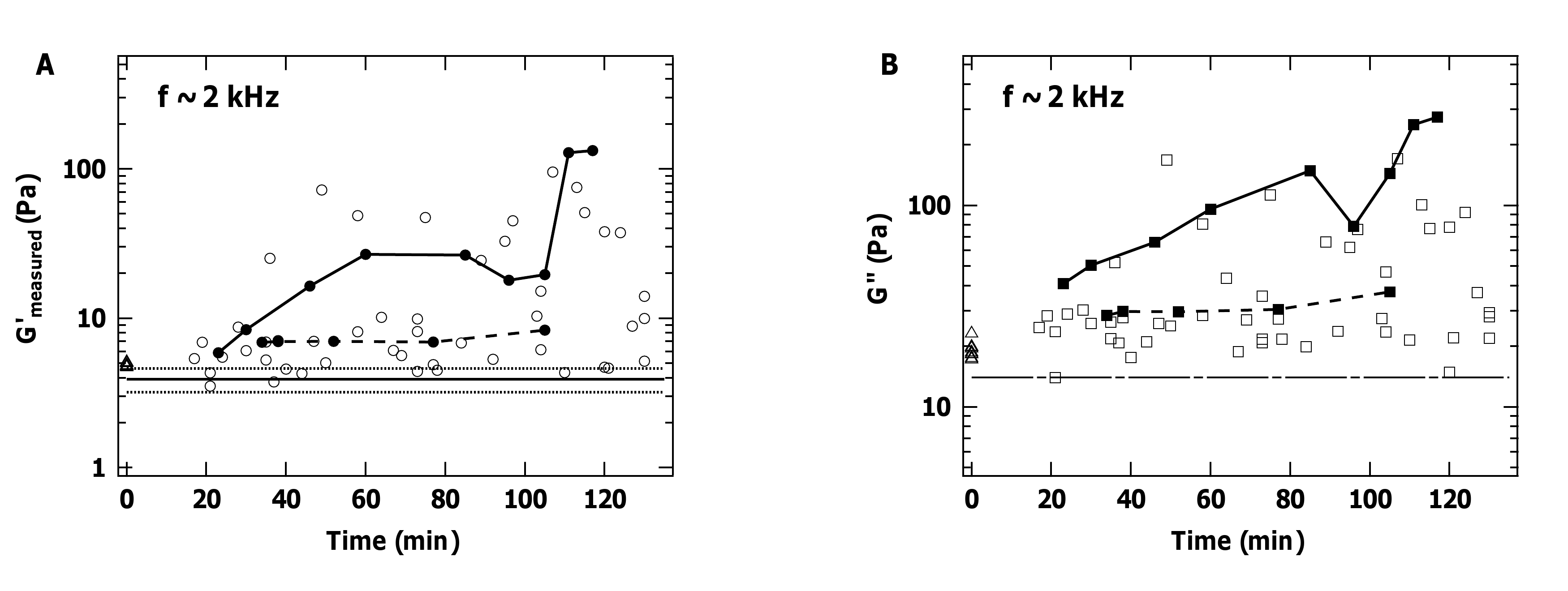

Supplement: Figure S5 — Time-dependent evolution of local viscoelasticity as collagen self-assembly proceeds. Measured (A) elastic and (B) viscous moduli in an assembling collagen fibrillar gel, reproduced from Figure 5. In rare circumstances, we were able to retain a given probe particle for measurements at multiple timepoints. These results are indicated by filled symbols, and are connected by lines to guide the eye. A significant evolution in viscoelastic properties is seen at one location (solid line), while in another location, no significant change is observed (dotted line). Further experiments would help to elucidate whether local moduli can both increase and decrease with time, or whether for example the transient decrease in moduli around 100 minutes is due to drift of the sample chamber. (TIF) [file pone.0070590.s005.tif]

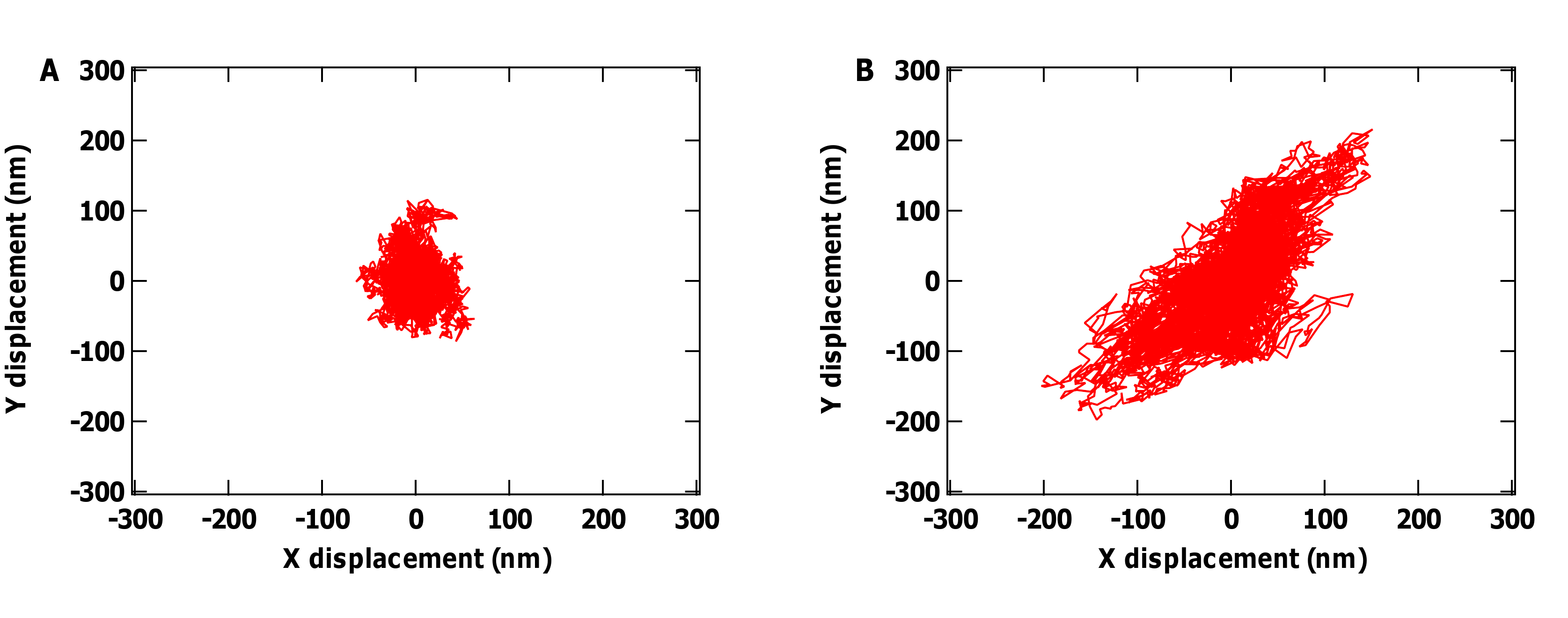

Supplement: Figure S6 — Heterogeneity of properties within a collagen gel. Plots here show the XY trajectories from Movie S1 of the (A) left bead and (B) right bead over ∼9 seconds. The motion of the left bead is more constrained (higher local elastic modulus), while the right bead exhibits substantial anisotropy of its motion. (TIF) [file pone.0070590.s006.tif]

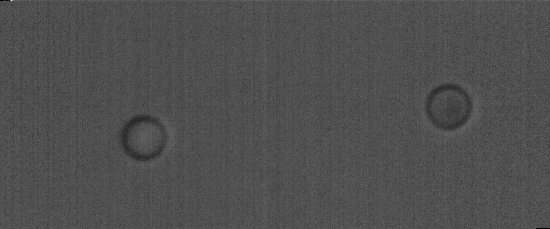

Supplement: Movie S1 — Heterogeneity of properties within a collagen gel. The movie shows the movement of two (non-trapped) beads diffusing in a collagen gel sample prepared at 0.5 mg/ml collagen concentration and at room temperature. (GIF) [file pone.0070590.s007.gif]
